# Bronchialkarzinom: Metastasierungspfade und Involvierung hilärer und mediastinaler Lymphknoten

**DOI:** 10.1007/s00117-022-01102-7

**Published:** 2023-01-02

**Authors:** Julian Glandorf, Jens Vogel-Claussen

**Affiliations:** Institut für Diagnostische und Interventionelle Radiologie, Medizinische Hochschule Hannover, Carl-Neuberg-Str. 1, 30625 Hannover, Deutschland

**Keywords:** Tumorerkrankungen, Lungenkarzinom, Stadieneinteilung, Mediastinale Lymphknotenstationen, Metastasierung, Neoplasms, Lung cancer, Staging, Mediastinal lymph node stations, Neoplasm metastasis

## Abstract

**Bedeutung des Lungenkarzinoms:**

Das Lungenkarzinom hat aufgrund seiner hohen Prävalenz und Mortalität enorme sozioökonomische Auswirkungen auf unsere Gesellschaft. Für das Jahr 2022 wurden ca. 59.700 Neuerkrankungen an Lungenkrebs prognostiziert.

**TNM-Schema zur Stadieneinteilung:**

Die korrekte Ausbreitungsdiagnostik ist für die Therapieplanung, Prognoseabschätzung und zukünftige Analysen von grundlegender Bedeutung. Die Stadieneinteilung des Lungenkarzinoms erfolgt nach dem TNM-Schema der Union for International Cancer Control (UICC). Für die Stadien IIB–IIIC wird der Lymphknotenbefall zur Differenzierung herangezogen.

**Lymphknotenstationen beim Lungenkarzinom:**

Die Kenntnis der intrathorakalen Lymphknotenstationen ist für die genaue Klassifikation von entscheidender Bedeutung, und deren Befall hat unmittelbare Auswirkungen auf die Therapie. Die International Association for the Study of Lung Cancer (IASLC) hat eine vereinheitlichte Lymphknotenkarte vorgeschlagen, die sich durch ihre exakten anatomischen Definitionen auszeichnet und in der aktuellen S3-Leitlinie für das Lungenkarzinom berücksichtigt wird. Der Lymphknotenbefall wird abhängig vom Ausmaß in N0–N3 stratifiziert. Je nach Lokalisation des Primärtumors lassen sich bevorzugte Metastasierungspfade nachweisen. Die Krankheitslast hat einen größeren Einfluss auf das Überleben als die Lokalisation der Metastasen.

**Ausbreitungsdiagnostik beim Lungenkarzinom:**

Mit der Computertomographie (CT) kann die Operabilität des Primärtumors meistens sicher beurteilt werden. Invasive Verfahren zur Diagnosesicherung durch Probengewinnung sollten erst im Anschluss an nichtinvasive Diagnostik eingesetzt werden.

**Empfehlung für die Praxis:**

In der aktuellen Leitlinie des Lungenkarzinoms wird bei allen Patienten mit nichtkleinzelligem Lungenkarzinom, die einer kurativen Resektion zugeführt werden, eine systematische Lymphknotenresektion empfohlen.

## Abstract (en)

**Significance of lung cancer:**

Lung cancer has enormous socioeconomical impact on our society due to its high prevalence and mortality. About 59,700 new cases of lung cancer were forecasted for 2022.

**TNM scheme for staging:**

Correct staging is the basis for therapy planning, prognosis estimation, and future analyses. Staging is performed using the TNM scheme from the Union for International Cancer Control (UICC). Involvement of lymph nodes is used to differentiate between stage IIB and IIIC.

**Lymph node levels for lung cancer:**

Knowledge of the intrathoracic lymph node levels is crucial for the exact classification and its involvement has direct implications on therapy. The International Association for the Study of Lung Cancer (IASLC) proposed a unified lymph node map with exact anatomic definitions, which is recommended by the German national lung cancer guideline. The extent of lymph node involvement is stratified into N0–N3. Different metastatic paths are known depending on the location of the primary tumor, but the burden of disease has a greater influence on survival, than the location of metastases.

**Assessing the spread of lung cancer:**

Computed tomography can assess operability of the primary tumor safely in most cases. Invasive procedures to confirm the diagnosis by sampling tissue should be performed after noninvasive diagnostics.

**Practical recommendation:**

Systematic lymph node dissection for all patients with non-small cell lung cancer intended for curative resection is recommended in the current German national guideline for lung cancer.

Das Lungenkarzinom hat aufgrund seiner hohen Prävalenz und Mortalität enorme sozioökonomische Auswirkungen auf unsere Gesellschaft. Die korrekte Ausbreitungsdiagnostik ist für die Therapieplanung, Prognoseabschätzung und zukünftige Analysen von grundlegender Bedeutung. Fokus dieses Artikels ist die mediastinale Ausbreitung mit der Rolle des N‑Deskriptors im aktuellen TNM-Schema der Union for International Cancer Control (UICC) und den Lymphknotenstationen der International Association for the Study of Lung Cancer (IASLC) sowie den typischen Metastasierungspfaden des Lungenkarzinoms.

## Epidemiologie des Lungenkarzinoms

Für das Jahr 2022 wurden von der Gesellschaft der epidemiologischen Krebsregister e. V. und vom Zentrum für Krebsregisterdaten ca. 59.700 Neuerkrankungen an Lungenkrebs prognostiziert. Mit ca. 58 % betrifft die Mehrheit dieser Fälle Männer. Während bei Männern allerdings seit Ende der 1980er Jahre die Inzidenz abnimmt, ist bei Frauen immer noch ein stetig steigender Trend mit aktuell ca. 25.000 Neuerkrankungen zu verzeichnen. Im Vergleich der häufigsten Tumorlokalisationen befindet sich das Lungenkarzinom hierdurch auf dem dritten Platz bei den Frauen und auf dem zweiten Platz bei den Männern. Neben der hohen Inzidenz ist auch die Mortalität des Lungenkarzinoms mit einer mittleren 5‑Jahres-Überlebensrate von 22 % bei den Frauen und 17 % bei den Männern hoch. Die Prognose ist im Einzelfall jedoch stark vom Krankheitsstadium bei Erstdiagnose abhängig. Diese hohe Prävalenz und Mortalität führen dazu, dass das Lungenkarzinom für beide Geschlechter zusammengefasst die häufigste Krebstodesursache darstellt [3].

Zigarettenrauch ist in Deutschland für ca. 85 % aller Fälle verantwortlich und stellt somit den überragenden Risikofaktor für Lungenkrebs dar. Das Risiko wird durch den Beginn und die Dauer des Rauchens oder Passivrauchens sowie der Quantität beeinflusst. Darüber hinaus gibt es eine Vielzahl an weiteren Risikofaktoren, die mit einer Steigerung des Lungenkrebsrisikos einhergehen. Hierzu zählt die Exposition gegenüber natürlicher oder zivilisatorischer Strahlung, verunreinigter Luft durch Feinstaub, Dieselabgasen, Asbest, künstlichen Mineralfasern, polyzyklischen aromatischen Kohlenwasserstoffen, Chromaten, Siliziumdioxid, Arsen, Nickel, Mono- und Dichlordimethylether, Beryllium, Wolfram- und Kobaltstaub und Cadmium [14].

## N-Deskriptor im TNM-Schema der UICC

Die Stadieneinteilung des Lungenkarzinoms erfolgt nach dem TNM-Schema der Union for International Cancer Control (UICC) und dient zur Abschätzung der Prognose, Auswahl der therapeutischen Optionen und retrospektiven Analyse der Behandlungsergebnisse. Sie umfasst neben der Ausbreitung des Primärtumors (T-Staging) auch den Befall von Lymphknoten (N-Staging) und Fernmetastasen (M-Staging) [16]. Die Krankheitsstadien sind so konzipiert, dass die Überlebensraten durch sie möglichst differenziert abgebildet werden.

**Figure 1:**
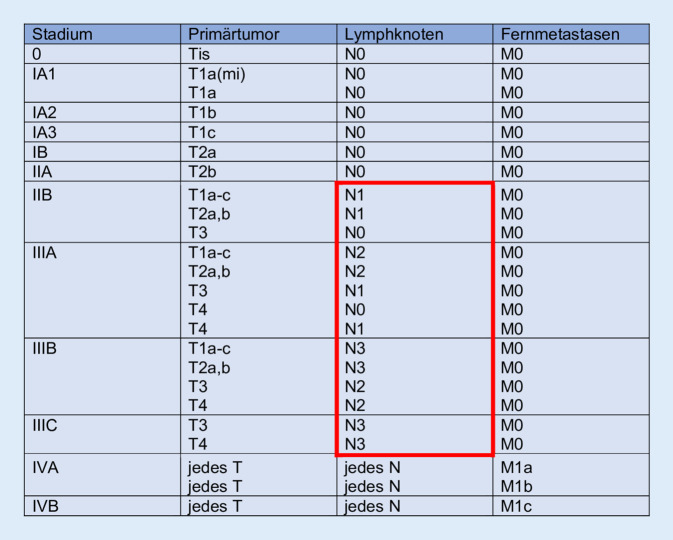

Die frühen Tumorstadien 0–IIA werden unter Abwesenheit von Lymphknoten oder Metastasen ausschließlich durch die Größe und Ausbreitung des Primärtumors definiert. Für die Stadien IIB–IIIC wird zusätzlich der Lymphknotenbefall zur Differenzierung herangezogen. Jegliches Vorhandensein von Metastasen führt zur Einteilung in die Stadien IVa–c (Abb. 1).

Vor diesem Hintergrund ist die Kenntnis der intrathorakalen Lymphknotenstationen für die genaue Klassifikation von entscheidender Bedeutung. Außerdem hat deren Befall unmittelbare Auswirkungen auf die Therapie. Hierzu wird eine Stratifizierung abhängig vom Ausmaß der lymphatischen Beteiligung in N0–N3 vorgenommen (Tab. 1).

**Table 1:** 

*N* x	Regionale Lymphknoten können nicht beurteilt werden
N0	Keine regionären Lymphknotenmetastasen
N1	Metastase(n) in ipsilateralen peribronchialen und/oder ipsilateralen Hilus- oder intrapulmonalen Lymphknoten (einschließlich eines Befalls durch direkte Ausbreitung des Primärtumors)
N2	Metastase(n) in ipsilateralen mediastinalen und/oder subkarinalen Lymphknoten
N3	Metastase(n) in kontralateralen mediastinalen, kontralateralen Hilus-, ipsi- oder kontralateralen Skalenus- oder supraklavikulären Lymphknoten

## Lymphknotenstationen gemäß der IASLC

**Figure 2:**
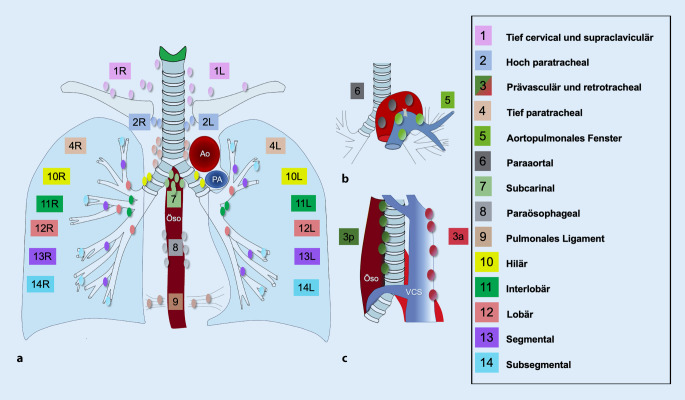

In der Vergangenheit haben sich die japanische Naruke-Karte und die Einteilung der American Thoracic Society nach Mountain-Dressler-Modifikation für die intrathorakalen Lymphknotenstationen etabliert [18, 19]. Aufgrund ihrer geringen Diskrepanzen, entstanden hierdurch häufig unterschiedliche Krankheitsstadien, die einen internationalen Vergleich der Krankheitsdaten erschwerten. In ihren Bestrebungen für eine internationale Krankheitsdatenbank, hat die International Association for the Study of Lung Cancer (IASLC) eine vereinheitlichte Einteilung vorgeschlagen [24]. Die IASLC-Lymphknotenkarte zeichnet sich durch ihre exakten anatomischen Definitionen aus und wird für die aktuelle S3-Leitlinie für das Lungenkarzinom berücksichtigt (Abb. 2).

Die einzelnen IASLC-Lymphknotenstationen werden im Folgenden beschrieben.

### Supraklavikuläre Zone

- Level 1 – tief zervikal, supraklavikulär und suprasternal
  - Obergrenze: Unterrand des Cartilago cricoidea
  - Untergrenze: Klavikula beidseits bzw. Manubrium sterni zentral
  - Die Mittellinie der Trachea unterteilt in 1R und 1L

### Obere mediastinale Zone

- *Level 2 – hoch paratracheal*
  - 2R&L – Obergrenze: Lungenspitze und Pleuraraum bzw. Obergrenze des Manubrium sterni
  - 2R – Untergrenze: Kreuzung des Unterrandes der Vena brachiocephalica mit der Trachea
  - 2L – Untergrenze: Oberrand des Aortenbogens
  - Der linke Rand der Trachea unterteilt in 2R und 2L
- *Level 3 – prävaskulär und retrotracheal*
  - 3a: prävaskulär
    Obergrenze: Spitze des Thorax
    Untergrenze: Carina
    Vordergrenze: Hinterrand des Sternums
    Hintergrenze rechts: Vorderrand der Vena cava superior
    Hintergrenze links: linke Arteria carotis
  - 3p: retrotracheal
    Obergrenze: Spitze des Thorax
    Untergrenze: Carina
- *Level 4 – tief paratracheal*
  - 4R – Obergrenze: Kreuzung des Unterrands der Vena brachiocephalica mit der Trachea
    Untergrenze: Untergrenze der Vena azygos
  - 4L: medial des Ligamentum arteriosum
    Obergrenze: Oberrand des Aortenbogens
    Untergrenze: Oberrand der linken Pulmonalarterie
    Der linke Rand der Trachea unterteilt in 4R und 4L

### Aortopulmonale Zone

- Level 5 – aortopulmonales Fenster
  - Subaortale Lymphknoten lateral des Ligamentum arteriosum
  - Obergrenze: Untergrenze des Aortenbogens
  - Untergrenze: Oberrand der linken Pulmonalarterie
- Level 6 – paraaortal
  - Lymphknoten anterior und lateral der Aorta ascendens und des Aortenbogens
  - Obergrenze: Tangente zum Oberrand des Aortenbogens
  - Untergrenze: Unterrand des Aortenbogens

### Subcarinale Zone

- Level 7 – subcarinal
  - Obergrenze: Carina
  - Untergrenze links: Oberrand des linken Unterlappenbronchus
  - Untergrenze rechts: Unterrand des Bronchus intermedius

### Untere Zone

- Level 8 – paraösophageal (außer subcarinal)
  - Obergrenze links: Oberrand des linken Unterlappenbronchus
  - Obergrenze rechts: Unterrand des Bronchus intermedius
  - Untergrenze: Diaphragma
- Level 9 – pulmonales Ligament
  - Obergrenze: Unterrand der Pulmonalvene
  - Untergrenze: Diaphragma

### Hiläre und interlobäre Zone

- Level 10 – hilär
  - Obergrenze rechts: Unterrand der Vena azygos
  - Obergrenze links: Oberrand der Pulmonalarterie
  - Untergrenze: Beidseits interlobär
- Level 11 – interlobär
  - Zwischen den Ursprüngen der Lappenbronchien
  - 11 superior: zwischen rechtem Oberlappenbronchus und Bronchus intermedius
  - 11 inferior: zwischen rechtem Mittel- und Unterlappenbronchus

### Periphere Zone

- Level 12 – lobär
  - Angrenzend an die Lappenbronchien
- Level 13 – segmental
  - Angrenzend an die Segmentbronchien
- Level 14 – subsegmental
  - Angrenzend an die Subsegmentbronchien

Die IASLC hat für zukünftige Analysen vorgeschlagen, die Lymphknotenlevel zur Steigerung der statistischen Signifikanz in 7 Level zu konsolidieren [24]. Eine weitere Stratifizierung abhängig vom Ausmaß der lymphatischen Beteiligung in N0–N3 erfolgt in der TNM-Klassifikation und differenziert nach Prognose (Tab. 1). Hierbei entsprechen Lymphknotenstationen mit zwei Ziffern (Level 10–14) der N1-Kategorie und Lymphknotenstationen mit einer Ziffer (Level 1–9) der N2-Kategorie.

## Mediastinale Metastasierungspfade des Lungenkarzinoms

**Figure 3:**
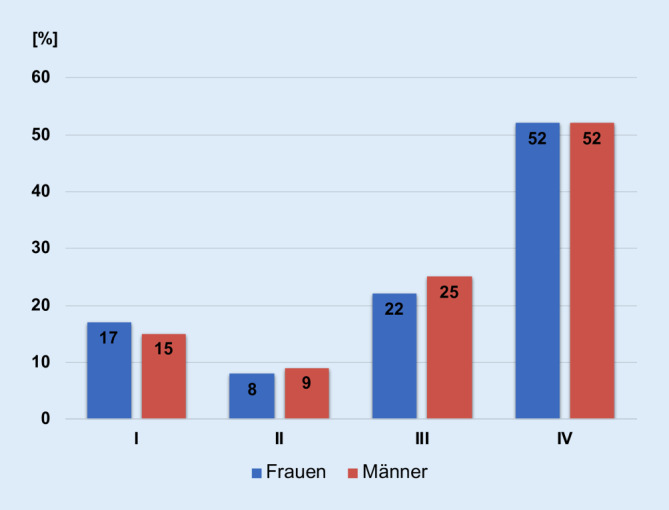

Die zumeist erst spät einsetzende Symptomatik führt dazu, dass Lungenkarzinome lange unerkannt bleiben und der überwiegende Anteil der Neuerkrankungen in den Krankheitsstadien III und IV diagnostiziert wird (Abb. 3). Somit liegen oftmals Lymphknotenmetastasen bei Diagnosestellung vor.

Die IASLC konnte in der Analyse ihrer Datenbank zeigen, dass Tumoren im linken Oberlappen am häufigsten N1- und N2-Metastasen aufwiesen. Außerdem konnte gezeigt werden, dass Lungenkarzinome im rechten Oberlappen mehrheitlich nach rechts paratracheal (Level 4R) und vom linken Oberlappen zumeist nach peri- und subaortal (Level 5/6) metastasieren. Ferner metastasierten Tumoren im Mittel- und beiden Unterlappen vermehrt nach subcarinal (Level 7), bevor sie nach paratracheal (Level 4) metastasierten [6, 25, 26]. Eine experimentelle Studie zeigte, dass in etwa einem Viertel der Fälle eine direkte mediastinale Drainage unter Umgehung der interlobären und hilären Lymphknotenstationen erfolgt [22]. Diese Metastasen werden auch als *Skip-Metastasen* („skip metastases“) bezeichnet und treten im Vergleich zu anderen Tumorarten vermehrt bei Adenokarzinomen auf [15].

Bezüglich der Prognose zeigte sich wie erwartet, dass Fälle mit multiplen N1-Metastasen eine signifikant schlechtere Prognose aufwiesen als Fälle mit einzelner N1-Metastase. Ebenso hatten Patienten mit Skip-Metastasen und einzelnen N2-Metastasen bessere Überlebensraten als Fälle mit multiplen N2-Metastasen oder mit N1- und N2-Metastasen [11, 25].

Während sich in einer japanischen Studie bei geringer mediastinaler Metastasierung (N0 und N1) keine unterschiedliche Prognose abhängig von der Seite des Primärtumors zeigte, hatten Patienten mit N2-Status durch ein Karzinom in der linken Lunge eine schlechtere Prognose als Patienten mit rechtsseitigem Tumor. Ferner wurden signifikant unterschiedliche Prognosen zwischen intralobärer und hilärer N1-Metastasierung, zwischen hilärer N1- und unterer mediastinaler N2-Metastasierung sowie zwischen unterer und oberer mediastinaler N2-Metastasierung berechnet [21].

Schlussfolgernd wurden von der IASLC drei Kategorien mit abfallender Prognose aufgestellt:

1. einzelne N1-Metastasen,
2. multiple N1- oder einzelne N2-Metastasen,
3. multiple N2-Metastasen.

Insgesamt ließ sich jedoch ableiten, dass die allgemeine Krankheitslast einen größeren Einfluss auf das Überleben hat als die alleinige anatomische Lokalisation der Metastasen [25].

## Bildgebende Diagnostik mediastinaler Lymphknoten beim Lungenkarzinom

In der Diagnostik und in der Stadieneinteilung des Lungenkarzinoms spielten bildgebende Verfahren eine zentrale Rolle. Die Thoraxübersichtsaufnahme stellt die Basisdiagnostik dar und kann erste Hinweise auf das Vorliegen eines Lungenkarzinoms oder Lymphadenopathie liefern [2, 12].

**Figure 4:**
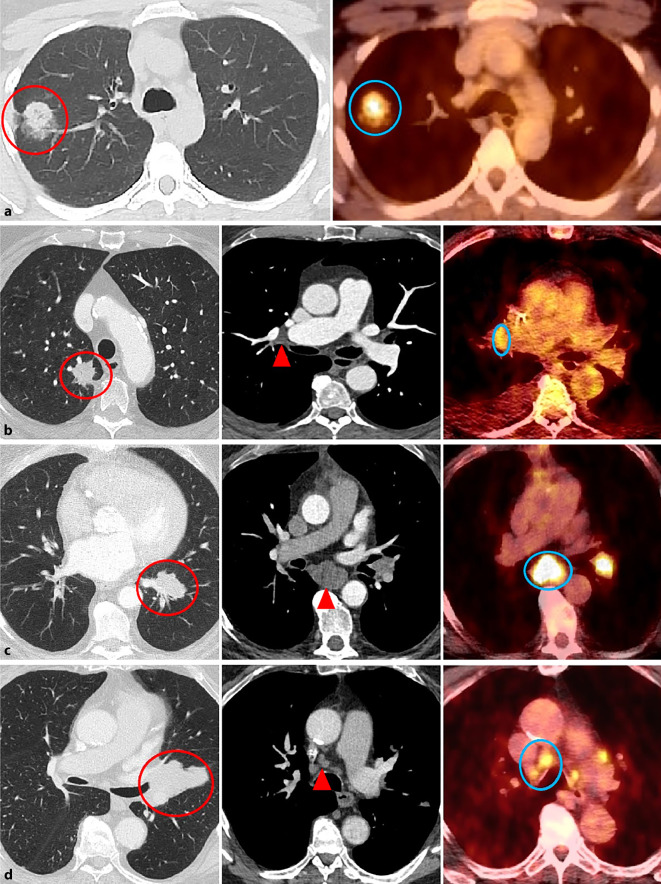

Sensitiver ist hingegen die Computertomographie (CT), mit der in der Regel die Operabilität des Primärtumors sicher beurteilt werden kann (Abb. 4; [17]). Hinsichtlich der lokalen Tumorausbreitung kann die Magnetresonanztomographie (MRT) wichtige Zusatzinformationen liefern. Hierbei ist insbesondere die Beurteilung einer Infiltration der Thoraxwand oder der mediastinalen Strukturen relevant. Außerdem ist sie zur Detektion bzw. zum Ausschluss von Hirnmetastasen die Methode der Wahl [14]. Auch in Bezug auf die lymphonodale und metastatische Ausbreitung stellt die CT die grundlegende Modalität dar.

Allerdings ist die Lymphknotengröße allein kein zuverlässiger Indikator einer Lymphknotenmetastasierung, da hierfür ebenso begleitende Infekte oder eine kardiale Dekompensation ursächlich sein können [17]. Andererseits können unauffällige Lymphknoten bereits Mikrometastasen enthalten. Ab 10 mm Kurzachsendurchmesser wird eine Sensitivität und Spezifität von ca. 62 % erreicht [13]. Entsprechend sollten sie im Befund gemäß der UICC-Klassifikation erwähnt werden, um im weiteren Verlauf abgeklärt werden zu können [14].

Zur genaueren Differenzierung vergrößerter Lymphknoten oder zur Identifikation von etwaigen Metastasen wird die Positronen-Emissions-Tomographie/Computertomographie (PET/CT) empfohlen. Diese nuklearmedizinische Ganzkörperuntersuchung füllt diagnostische Lücken und weist eine sehr hohe Sensitivität und Spezifität auf (Abb. 4, Fusionsbilder; [4, 5, 20, 28]).

Invasive Verfahren zur Diagnosesicherung durch Probengewinnung von Lymphknoten sollten erst im Anschluss an nichtinvasive Diagnostik eingesetzt werden. Die Bronchoskopie stellt hierbei die wichtigste Methode dar. Der mediastinale Lymphknotenstatus sollte mithilfe von endoskopischen Verfahren wie der Bronchoskopie ultraschallgesteuert biopsiert und pathologisch erfasst werden. Bei peripher gelegenen Rundherden sollten transthorakale bildgesteuerte Biopsien erfolgen [14].

## Therapie des Lungenkarzinoms im Hinblick auf mediastinale Lymphknoten

In der aktuellen Leitlinie des Lungenkarzinoms wird bei allen Patienten mit nichtkleinzelligem Lungenkarzinom, die einer kurativen Resektion zugeführt werden, eine systematische Lymphknotenresektion empfohlen [14]. Dieser Empfehlung liegen zahlreiche Studien zugrunde, die einen signifikanten Überlebensvorteil nach systematischer Lymphknotenresektion gegenüber einer mediastinalen Lymphknotensammlung zeigten [8, 9, 27, 29, 30].

Ferner konnte durch die systematische Lymphknotenresektion ein genaueres N‑Staging durch eine signifikant häufigere Detektion eines N2-Multilevel-Status erreicht werden [7, 10]. Es wurden allerdings auch mehr spezifische Komplikationen und eine längere Operationsdauer durch die systematische Lymphknotenresektion nachgewiesen [9, 27]. Diese sind in der Regel jedoch gut beherrschbar, sodass hierdurch keine erhöhte Letalität entsteht [8, 30].

Besondere Bedeutung hat der mediastinale Metastasierungsstatus in der sehr heterogenen Gruppe im UICC-Stadium III. Dieses Stadium umfasst Tumoren von T1–T4 und wird nochmals in IIIA–C unterteilt. Dass der N2-Status verschiedene Subgruppen umfasst, konnten Andre et al. zeigen, indem sie für mikroskopischen Befall eines einzelnen und mehrerer Level sowie den computertomographischen Befall eines einzelnen sowie mehrerer Level unterschiedliche 5‑Jahres-Überleben aufzeigen konnten [1]. Daher wird in der S3-Leitlinie die Verwendung der Robinson-Klassifikation für die Unterteilung des Stadiums IIIA mit N2-Status empfohlen (Tab. 2; [14, 23]).

**Table 2:** 

Untergruppe des Stadiums IIIA (N2)	Beschreibung
IIIA_1_	Inzidenteller Nachweis von mediastinalen Lymphknotenmetastasen in einer Lymphknotenstation bei der postoperativen histologischen Untersuchung des Resektats
IIIA_2_	Intraoperativer Nachweis von Lymphknotenmetastasen in einer Lymphknotenstation
IIIA_3_	Präoperativer Nachweis von Lymphknotenmetastasen in einer oder mehreren Lymphknotenstationen durch Staging mittels Mediastinoskopie, Feinnadelbiopsie oder PET
IIIA_4_	„Bulky“ (ausgedehnte) oder fixierte N2-Metastasen oder Metastasen in mehreren Lymphknotenstationen (mediastinale Lymphknoten > 2–3 cm mit extrakapsulärer Infiltration; Befall mehrerer N2-Lymphknotenpositionen; Gruppen multipler befallener kleinerer (1–2 cm) Lymphknoten)

## Fazit für die Praxis

- Die Stadieneinteilung des Lungenkarzinoms erfolgt nach dem TNM-Schema der Union for International Cancer Control (UICC) und dient zur Abschätzung der Prognose, Auswahl der therapeutischen Optionen und retrospektiven Analyse der Behandlungsergebnisse.
- Mediastinale Lymphknoten sollten gemäß der IASLC-Karte beschrieben und mittels Positronen-Emissions-Tomographie/Computertomographie (PET/CT) oder endoskopischer Biopsie weiter abgeklärt werden.
- Im TNM-Schema wird in N0–N3 mit unterschiedlicher Prognose stratifiziert.
- Lungenkarzinome im rechten Oberlappen metastasieren mehrheitlich nach rechts paratracheal (Level 4R); vom linken Oberlappen zumeist nach peri- und subaortal (Level 5/6); vom Mittel- und beiden Unterlappen vermehrt nach subcarinal (Level 7), bevor sie nach paratracheal (Level 4) metastasierten.
- In etwa einem Viertel der Fälle treten *Skip-Metastasen* auf.
- In der aktuellen Leitlinie des Lungenkarzinoms wird bei allen Patienten mit nichtkleinzelligem Lungenkarzinom, die einer kurativen Resektion zugeführt werden, eine systematische Lymphknotenresektion empfohlen.
